# Ball Milling Improves Physicochemical, Functionality, and Emulsification Characteristics of Insoluble Dietary Fiber from *Polygonatum sibiricum*

**DOI:** 10.3390/foods13152323

**Published:** 2024-07-24

**Authors:** Jingxuan Ke, Xin Wang, Xinyu Gao, Yuhui Zhou, Daqing Wei, Yanli Ma, Cuicui Li, Yilin Liu, Zhizhou Chen

**Affiliations:** 1 Zhang Zhongjing School of Chinese Medicine, Nanyang Institute of Technology, Nanyang 473004, China; jingxke@163.com (J.K.); 17550309142@163.com (X.G.); 19639783660@163.com (Y.Z.); bai23677a@163.com (D.W.); licui8@yeah.net (C.L.); 15236286951@163.com (Y.L.); 2 College of Food Science and Technology, Hebei Agricultural University, Baoding 071000, China; m17732941206@163.com (X.W.); chenzhizhou2003@126.com (Z.C.)

**Keywords:** *Polygonatum sibiricum*, insoluble dietary fiber, ball milling, physicochemical and functional properties, Pickering emulsion

## Abstract

The effects of ball milling on the physicochemical, functional, and emulsification characteristics of *Polygonatum sibiricum* insoluble dietary fiber (PIDF) were investigated. Through controlling milling time (4, 5, 6, 7, and 8 h), five PIDFs (PIDF-1, PIDF-2, PIDF-3, PIDF-4, and PIDF-5) were obtained. The results showed that ball milling effectively decreased the particle size and increased the zeta-potential of PIDF. Scanning electron microscope results revealed that PIDF-5 has a coarser microstructure. All PIDF samples had similar FTIR and XRD spectra. The functional properties of PIDF were all improved to varying degrees after ball milling. PIDF-3 had the highest water-holding capacity (5.12 g/g), oil-holding capacity (2.83 g/g), water-swelling capacity (3.83 mL/g), total phenol (8.12 mg/g), and total flavonoid (1.91 mg/g). PIDF-4 had the highest ion exchange capacity. Fat and glucose adsorption capacity were enhanced with ball milling time prolongation. PIDF-5 exhibited a contact angle of 88.7° and lower dynamic interfacial tension. Rheological results showed that PIDF-based emulsions had shear thinning and gel-like properties. PE-PIDF-5 emulsion had the smallest particle size and the highest zeta-potential value. PE-PIDF-5 was stable at pH 7 and high temperature. The findings of this study are of great significance to guide the utilization of the by-products of *Polygonatum sibiricum*.

## 1. Introduction

*Polygonatum sibiricum* is an important medicinal and edible homology plant in China [1]. It contains various active ingredients and has significant pharmacological effects, such as regulating immunity and hypotensive and hypoglycemic effects [2]. Besides polysaccharides and saponins, insoluble dietary fiber (IDF) is the main functional component in *Polygonatum sibiricum* [3]. The root tuber of *Polygonatum sibiricum* is rich in dietary fiber, with the IDF content ranging from 88.12% to 92.90% [3]. After polysaccharide extraction, the *Polygonatum sibiricum* residue still contains a lot of dietary fiber, which has potential application in functional food.

IDF can hold water and oil and stabilize the water interface. Therefore, it can be applied to Pickering emulsions as a solid particle emulsifier. Pickering emulsion plays an important role in the food industry because of its high stability, green preparation method, and wide application range. In recent years, the use of natural biomacromolecule particles to construct edible, safe, and environmentally friendly Pickering emulsion has become a research hotspot in food production. Our research group’s previous studies showed that *Polygonatum sibiricum* insoluble dietary fiber (PIDF) extracted by an ultrasonic-assisted enzyme method had a higher yield [4]. However, the extracted PIDF has coarse particles and strong hydrophilicity, which limits its application in Pickering emulsion. Reduction of particle size could improve the functional properties of IDF (e.g., water-holding capacity, oil-holding capacity, swelling power, cation exchange capacity, oil and glucose adsorption capacity, etc.) [5]. IDF can be modified by enzymatic, chemical, physical, and other methods [6,7,8]. A physical modification method has the advantages of simple operation and being green, pollution-free, and so on [9]. Among them, ball milling can effectively reduce particle size and increase the specific surface area of IDF. Previous researchers [10] reported that ball milling of pomelo (*Citrus grandis*) peel (PP-IDF) to submicron scale effectively enhanced its water- and oil-holding capacity and water-swelling capacity, and ball-milled PP-IDFs successfully prepared Pickering emulsions (O/W). Other researchers [11] reported applied ball milling on three citrus fibers and demonstrated particle size reduction by ball milling combined with enhanced physicochemical properties. Therefore, PIDF was also treated by ball milling modification in this study.

There are few reports about extraction, characterization, and application in the functional emulsion of IDF obtained from *Polygonatum sibiricum*. Therefore, this study aimed to investigate the effect of ball milling modification on the physicochemical and functional properties of PIDF. First, modified PIDFs were prepared using different milling times. Next, the physicochemical, micromorphological, functional, and emulsification properties and the properties of Pickering emulsions of the five modified PIDFs were investigated. Exploring the modification methods and characteristics of PIDF is conducive to expanding the application range of *Polygonatum sibiricum* in the food and health products industry, providing a reference for the rational development of *Polygonatum sibiricum* resources.

## 2. Materials and Methods

### 2.1. Materials and Reagents

*Polygonatum sibiricum* was purchased from Henan Lianyuan Biotechnology Co., Ltd., Nanzhao, China. The *Polygonatum sibiricum* residue, a waste product after the polysaccharide was extracted (hot water extraction), was from our laboratory. The *Polygonatum sibiricum* residue was dried at 55 °C (101-4A, Shanghai Jiecheng Experimental Instrument Co., Ltd., Shanghai, China) for 48 h, crushed, passed through a 60-mesh sieve, and stored in a glass desiccator for later use. Peanut oil was purchased from Cofco Fulinmen Food Marketing Co., Ltd., Rizhao, China. α-amylase (1000 U/g) was purchased from Shandong Longcott Enzyme Preparation Co., Ltd., Linyi, China. Papain (100,000 U/g) was purchased from Nanning Pangbo Biological Engineering Co., Ltd., Nanning, China. The experimental water was deionized water.

### 2.2. Preparation of PIDF

PIDF was obtained from *Polygonatum sibiricum* residue using our previously optimized method [4]. In brief, *Polygonatum sibiricum* residue powder was fully mixed with deionized water at a solid/liquid ratio of 1:20. Then, the mixture was adjusted to pH 6 and placed in a water bath at 60 °C, undergoing enzymatic hydrolysis by α-amylase (0.25%) for 1 h. Next, the pH of the mixture was adjusted to 5, and papain (0.125%) was added, which was incubated at 50 °C for 1 h. Then, the mixture was boiled to inactivate all enzymes. After enzymolysis, the mixture was placed in an ultrasonic cell crusher (Ningbo Xinzhi Biotechnology Co., Ltd., Ningbo, China) and underwent ultrasonic treatment at 50 °C for 20 min (ultrasonic 2 s and intermittent 2 s). Upon cooling to room temperature, the mixture was centrifuged at 4000 rpm for 10 min. The residue was washed twice with deionized water, then washed once with 75% ethanol solution, and finally dried to constant weight, recorded as PIDF. PIDF consists mainly of cellulose (24.4%), hemicellulose (31.5%), lignin (20.8%), protein (7.5%), starch (4.1%), and ash (0.97%) [4].

### 2.3. Ball Milling Treatment

PIDF was treated with a planetary ball mill (CJM-SY-B, Qinhuangdao Bomao New Material Technology Co., Ltd., Qinhuangdao, China) at 380 rpm for 4, 5, 6, 7, and 8 h [12]. The grinding medium is a zirconia ball, and the temperature is less than 20 °C. The processed powders were designated PIDF-1, PIDF-2, PIDF-3, PIDF-4, and PIDF-5.

### 2.4. Characterization of Milled PIDF

#### 2.4.1. Particle Size Analysis

The particle size distribution of the five kinds of PIDF was determined by a laser particle size analyzer (Rise-2006, Jinan Runzhi Technology Co., Ltd., Jinan, China). Distilled water was used as the dispersion medium, and the pump speed was 20. The PIDFs were scattered into the sample pool, and the experimental data were recorded when the refractive index reached 1.33 [13].

#### 2.4.2. Zeta-Potential

The zeta-potential of the five kinds of PIDF (0.1%) was measured using ZETASIZER NANO ZSEs (Malvern Instruments Ltd., Malvern, UK) at 25 °C. The balance time was 120 s [14].

#### 2.4.3. Fourier Transform Infrared Spectroscopy (FTIR)

The five PIDFs’ FTIR spectra were detected using an FTIR instrument (Spectrum TWO DTGS, PerkinElmer, Norwalk, CT, USA) with a KBr disk as the background [15]. The FTIR spectral data were collected in a range of 500–4000 cm^−1^ at 32 scans with a resolution of 0.5 cm^−1^.

#### 2.4.4. X-ray Diffraction (XRD)

The five PIDF particles were scanned by X-ray diffraction (MininFlex 600, Rigaku, Akishima, Japan). The determination conditions were as follows: Cu-Kα target (λ = 0.154 nm), voltage 40 kV, current 25 mA, scanning range from 5 to 80°, scanning speed at 10°/min, and step size 0.05°.

#### 2.4.5. Scanning Electron Microscopy (SEM)

The micrograph was observed using an SEM (JSM-7900F, JEOL, Akishima, Japan) at 2.0 KV. The sample was dispersed on the conductive adhesive and sprayed with gold. The images were observed at 500× and 5000×.

### 2.5. Functional Properties

#### 2.5.1. Water-Holding Capacity (WHC), Oil-Holding Capacity (OHC) and Water-Swelling Capacity (WSC)

WHC and OHC were analyzed using the method of reference [16]. The five kinds of PIDF (1.0 g) were mixed with 10 g of deionized water (or peanut oil) and stirred. Then, the resultant mixtures were centrifuged at 4000 rpm for 10 min. The WHC and OHC were calculated as grams of adsorbed or entrapped deionized water (peanut oil) per gram of PIDF (dry basis), according to the following formula:

WHC (OHC) = m_i_/m_o_ × 100% (1)

where m_i_ is the weight of PIDF after centrifugation and m_o_ is the weight of PIDF.

WSC was determined according to the procedure described in [17]. One gram of PIDF was hydrated in 15 mL of distilled water in a graduated test tube, oscillated evenly, and held at room temperature for 24 h. The bed volume occupied by the particles was recorded. WSC was calculated by the relative ratio of change in volume of PIDF before and after the hydration.

#### 2.5.2. Total Phenolics Content (TPC) and Total Flavonoid Content (TFC)

TPC and TFC of the five PIDFs were estimated using the Folin Ciocalteu method and the aluminum chloride colorimetric assay proposed by [18]. First, 0.5 g of PIDF was placed in an Erlenmeyer flask, 10 mL of 60% ethanol was added, and magnetic stirrer extraction was applied for 60 min. The mixture was centrifuged at 4000 r/min for 15 min, and the supernatant was further analyzed. For TPC determination, sample solution (1 mL) and standard gallic acid (10, 20, 30, 40, 50, and 60 μg/mL) were placed in a test tube, and 5 mL distilled water and 0.5 mL of Folin Ciocalteu reagent were added, and the mixture was shaken well. After 5 min, 1 mL of 7.5% (*v*/*v*) sodium carbonate was added, and distilled water to 10 mL. The absorbance was measured at 750 nm at room temperature for 2 h.

For TFC determination, a sample (1.0 mL) and 0.2 mg/mL rutin standard solution (0, 1, 2, 3, 4, and 5 mL) were tested. Then, 1.5 mL of 5% NaNO_2_ solution, 1.5 mL of 10% Al(NO_3_)_3_ solution, and 15 mL of 4% NaOH solution were added successively, mixed evenly, and then 70% ethanol was added to 25 mL. Absorbance was measured at 508 nm, and a standard curve was drawn.

#### 2.5.3. Antioxidant Ability

The antioxidant activity of the five kinds of PIDF was detected in scavenge 1, 1-diphenyl-2-picrylhydrazyl (DPPH) radicals following the method of [19]. In brief, PIDFs were prepared with anhydrous ethanol at concentrations of 0.5, 1.0, 1.5, 2.0, and 2.5 mg/mL. DPPH solution with a concentration of 0.2 mmol/L was prepared using anhydrous ethanol. Two mL PIDF solution with different concentration gradients was added into a 10 mL brown reagent tube, and then 2 mL DPPH solution was added. After mixing, the absorbance was measured at 517 nm after reaction for 30 min.

#### 2.5.4. Cation Exchange Capability (CEC)

CEC was determined using the method described by [15] with some modifications. One gram of PIDF was mixed with 10 mL hydrochloric acid (0.1 M) for 24 h at 4 °C. The residue was washed with deionized water and titrated with a silver nitrate solution until no white precipitate appeared. Then, it was dried in an oven at 50 °C for 6 h. After that, 0.25 g of residue was mixed with 100 mL of NaCl (5%) and titrated with 0.1 mol/L of NaOH solution. The calculation was made by recording the volume of NaOH solution consumed. Distilled water was used instead of HCl to determine the blank sample. CEC of the sample was calculated using the following formula:

CEC(mmol·g^−1^) = [(V_2_ − V_1_) × 0.1]/m (2)

where V_2_ is the volume of NaOH solution consumed by the sample (mL), and V_1_ is the volume of NaOH solution consumed by the blank sample (mL).

#### 2.5.5. Fat and Glucose Adsorption Capacity

Cholesterol adsorption capacity (CAC), sodium cholate adsorption capacity (SCAC), and glucose adsorption (GAC) were measured according to the method of [20,21,22] with some modifications. For CAC, fresh egg yolks were whisked with 9 times the volume of deionized water, 1 g of PIDF was added, and the mixture was stirred at 220 r/min for 2 min. It was adjusted to pH 2.0 and pH 7.0, respectively. The mixture was incubated in a shaker at 37 °C for 150 min (150 r/min). The mixture was then centrifuged at 4000 r/min for 30 min to collect the supernatant. The supernatant was diluted by 15 times. Finally, the cholesterol concentration was determined by the phthalaldehyde method. For SCAC, 1 g of PIDF was mixed with 100 mL sodium cholate (1 mg/mL) and adjusted to pH 7.0. The mixture was shaken in a 37 °C water bath (180 r/min) for 120 min. One mL reaction solution was sucked out every 20 min and centrifuged at 4000 r/min for 25 min. The supernatant was collected, and the concentration of sodium cholate was determined by the furfural colorimetric method. For GAC, 1 g of PIDF was mixed with 100 mL of glucose solution (20, 40, 60, 80, and 100 mg/mL), respectively. The mixtures were incubated in a shaker at 37 °C (160 r/min) for 180 min and centrifuged at 4000 r/min for 15 min. The supernatant was collected, and the glucose content was determined by the phenol–sulfuric acid method.

### 2.6. Emulsification Characteristics

#### 2.6.1. Contact Angle Analysis

The contact angle of the five kinds of PIDFs was measured by the Contact Angle Measuring Instrument (DSA100, KRUSS, Hamburg, Germany). PIDF particles were laid stably on the test table, and 2 μL liquid droplets were squeezed out with the microsyringe provided with the instrument, slowly lowering the needle, and then slowly lifting the needle when the droplets just contacted the base material [23]. A high-speed optical camera recorded the droplet’s shape, and the accurate value of the contact angle was obtained by contour fitting of the droplet with software.

#### 2.6.2. Dynamic Interfacial Tension (IFT)

The IFT at the oil–water interface of the five kinds of PIDF particles was measured at room temperature using a surface tension analyzer (Sigma 701, Sigma-Biolin, Gothenburg, Finland) equipped with standard platinum sheets (1.5 mm) and standard containers (25 mm). Before each measurement, the platinum plate was burned with a blue flame and then cooled. The calibration factor of soybean oil was measured in a small container (50 mm), and then 30 mL of soybean oil was carefully layered over 30 mL of water or PIDF dispersion in a standard container and left to rest for 10 min at 25 °C. The IFT of PIDF particles was measured at 60 min. The interfacial tension of the two immiscible solutions at the interface was equivalent to the maximum force required to pull the platinum plate from one phase to another [12].

#### 2.6.3. Pickering Emulsion Preparation

The oil-in-water Pickering emulsion stabilized by the five PIDFs was obtained according to [10]. Firstly, PIDF solutions (0.2 wt%, 80 mL) with 20 mL of peanut oil (20 wt% oil phase) were high-speed homogenized at 12,000 rpm for 3 min. Then, the first emulsion was intensified via a high-pressure homogenizer machine (APV-60, Shanghai Shun Yi Experimental Equipment Co., Ltd., Shanghai, China) at 40 MPa for three cycles.

#### 2.6.4. Zeta-Potential and Particle Size and Measurements

The zeta-potential of the Pickering emulsion was determined at a 20 times diluted concentration (0.05%, *w*/*w*) using ZETASIZER NANO ZSEs (Malvern Instruments Ltd., UK) at 25 °C [14]. The droplet size of the Pickering emulsion was determined using a Mastersizer 3000 (Malvern Instruments Ltd., UK), and the D_4,3_ and D_3,2_ were recorded.

#### 2.6.5. Microstructure of Pickering Emulsions

The microstructures of prepared Pickering emulsions were observed with an optical microscope (CX31, Olympus, Metropolitan Tokyo, Japan) with the Mshot micro digital imaging system (MD-50, Mshot, Guangzhou, China) under a 10× eyepiece and a 10× objective lens. The fresh emulsion was diluted 10 times with deionized water and placed on a slide, then covered with a coverslip [24].

#### 2.6.6. Rheological Properties

The rheological properties of the emulsions were measured by a rheometer (Discoveny HR-2, TA Instruments, New Castle, DE, USA) at 25 °C [25]. The freshly prepared emulsions were dripped into the middle of a 40 mm steel parallel plate with a gap of 1 μm. The apparent viscosity of Pickering emulsions evolved with a share rate (0.1–100 s^−1^) at 25 °C under the steady shear flow model. The strain scan (0.01–100%) was performed when the frequency was set at 1 Hz, and the strain variation range was determined. Frequency sweeps were performed to obtain storage modulus (G′) and loss modulus (G″) at a specific strain of 0.1% (within the linear viscoelastic region), varying in frequency from 0.1 to 100 rad·s^−1^ [26].

#### 2.6.7. Turbiscan Stability Index (TSI)

The freshly prepared emulsion was loaded into a special sample bottle and scanned at 55 °C for 6 h using a multi-light scattering stability analyzer (Turbiscan Lab, Formulaction, Toulouse, France). TurbiSoft-Lab-2.2.0.82-5 software was used to analyze the results, and the TSI was used to characterize the emulsion’s stability.

#### 2.6.8. Stability of Pickering Emulsions

To evaluate the storage stability of emulsions, 15 mL of Pickering emulsion was placed in a glass tube at room temperature (25 °C) for 30 d. Photographs were taken at 1, 15, and 30 days. Meanwhile, the height of the serum layer was measured. Creaming indexes (CIs) were calculated according to the following equation: Equation (3) [13]:

CI (%) = H_s_/H_e_ × 100% (3)

where H_e_ is the height of the total emulsion, and H_s_ is the height of the serum layer.

Ten mL of Pickering emulsion was placed in a test tube. Then, the emulsion was placed under constant temperature conditions of 25, 50, and 80 °C for 1 h. Photographs and CIs were recorded.

The pH values of the Pickering emulsions were adjusted to 3, 5, 7, and 9. Then, 10 mL of the emulsion was placed in a glass tube and stored at room temperature for 1, 5, 7, 15, 28, and 30 days to observe its stability. Photographs and CIs were recorded.

### 2.7. Statistical Analyses

The results were analyzed statistically using SPSS 27.0 (Chicago, IL, USA) by a one-way analysis of variance (ANOVA). ORIGIN 2021 (Minneapolis, MN, USA) was used for plotting. All tests were repeated in triplicate. Each result was presented as the mean ± standard deviation.

## 3. Results

### 3.1. Structure and Physicochemical Analysis

#### 3.1.1. Particle Size Distribution

**Figure 1:**
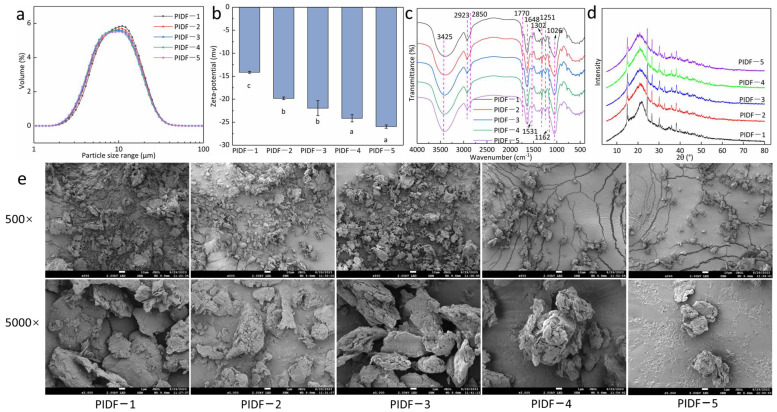
Characterization of milled PIDF. (**a**): particle size; (**b**): Zeta−potential; (**c**): FTIR spectra; (**d**): XRD spectra; (**e**): SEM images. Different lowercase letters indicate significant differences (*p* < 0.05).

It has been reported that ball milling can break larger particles into submicron particles, which can be more easily incorporated into food with other raw materials [18]. The particle size distribution of PIDF treated with different milling times is shown in Figure 1a. Consistent with the literature reports [18,27,28], long-term ball milling reduced particle size. The Dav (average particle size) of the five kinds of PIDF was 10.44 ± 0.06, 10.21 ± 0.02, 10.06 ± 0.02, 9.83 ± 0.05, and 9.96 ± 0.04 μm, respectively (*p* < 0.05).

The results show that the particle size of the five kinds of PIDF reached the submicron level, indicating that they can be used as a dietary fiber component in dietary fiber-fortified foods [18]. When the milling time was 8 h, the particle size of PIDF-5 did not decrease but increased, which may be due to aggregation between particles. When the particle size is too small, the specific surface area is large, and agglomeration can easily occur among particles [29]. The authors of [30] showed that the long-term milling process reduced the particle size and increased the van der Waals force and electrostatic force between the particles, resulting in the agglomeration of fine particles. In other references [27,28], no increase in particle size caused by prolonged milling time was found, possibly because the particle size was not small enough. In addition, the particle size during ball milling depends on conditions such as material type and speed [11].

#### 3.1.2. Zeta-Potential Analysis

The effects of different milling times on PIDF zeta-potential are shown in Figure 1b. The results show that the absolute value of PIDF increased with milling time. This was because in the process of grinding with zirconia balls, shear force and heat were generated to promote the formation of carboxyl groups on the cellulose surface [10]. Therefore, the zeta-potential was increased. When the milling time was 7 and 8 h, the absolute value of zeta-potential was the largest (−24.15 ± 0.77 mV and −25.95 ± 0.35 mV, respectively). The authors of [16] have also reported similar changes in the zeta-potential of okara after planetary ball milling (an increase from −9.5 mV to −22.7 mV). Additionally, the authors of [7] revealed that planetary ball milling enhanced the zeta-potential of citrus fiber.

#### 3.1.3. FTIR Analysis

The FTIR spectra of the five kinds of PIDF modified using different milling times are shown in Figure 1c. The results show that the FTIR spectra of the five kinds of PIDF showed the typical absorption characteristics of dietary fiber. The wide peaks near 3425 cm^−1^ are due to stretching vibrations of –OH. With the extension of milling time (especially for PIDF-4 and PIDF-5), the stretching vibration peak of –OH was sharper and higher, indicating that the modification of ball milling significantly enhanced the vibration peak of –OH, which may be related to the increase of the specific surface of the particles and the exposure of hydrogen bonds. The exposure of hydrogen bonds may affect the functional characteristics of dietary fiber, such as hydration capacity [31]. It has been reported that ball milling increased the hydrogen bond absorption intensity in the FTIR spectrum of sea buckthorn seed meal insoluble dietary fiber [15]. The absorption peak at 2923 cm^−1^ was generated by the asymmetric stretching vibration of the methylene C–H bond of polysaccharide [16]. The small peak at 2850 cm^−1^ is the asymmetric stretching vibration peak of the branched methylate (–CH_2_). The same absorption peak was observed in the infrared spectra of IDF from three-grain bran modified by ball milling [32].

The absorption peaks near 1770 cm^−1^ and 1648 cm^−1^ were formed by C=O stretching vibrations in COOH of aldehydic acid and polyphenols present in lignin or hemicelluloses [1]. The characteristic absorption peak around 1531 cm^−1^ of aromatic benzene in lignin was found in all samples [33]. The peak at 1302 cm^−1^ was sharper, which suggested the bending vibration peak of methyl C–H in sugar [32]. 1251 cm^−1^ was caused by C–H bending vibrations in hemicelluloses [6]. The wide absorption band near 1026 cm^−1^ was mainly due to the stretching of C–O, especially related to the glycosidic bond (C–O–C) [32]. In summary, the ball milling modification in this study did not change the types of functional groups in dietary fiber but enhanced the peak strength of the hydrogen bond.

#### 3.1.4. XRD Analysis

Effects of ball milling time on the crystallinity changes of PIDF are exhibited in Figure 1d. According to [34], the IDF constitutes the crystalline region (predominantly cellulose), while the non-crystalline region consists of hemicellulose, lignin, and some amorphous cellulose. The diffraction patterns of the PIDF obtained at different milling times showed a similar diffraction pattern, which exhibited a typical cellulose form. The major 2θ peak was observed around 14.96°, 22.06°, 24.36°, and 26.64°, which is typical of cellulose type I crystal structure, which suggests that both crystalline and amorphous regions coexist [6]. The irregular peaks in the diffraction patterns could be attributed to the destruction of the cellulose structure caused by the intense extraction conditions during the extraction process [30]. Similar XRD patterns were observed for citrus fibers [7] and sea buckthorn seed meal IDF [15].

With the extension of milling time, the particle size of dietary fiber was decreased, and the intensity of the main peaks (14.96°, 22.06°, 24.36°, and 26.64°) reduced (Figure 1d). This agrees with previous studies stating that micronization treatment can decrease the amorphous area of IDFs, such as okara IDF [30], sea buckthorn seed meal IDF [15], and Japanese grape (*Hovenia dulcis*) pomace IDF [35]. When the milling time exceeded 4 h (PIDF-2 (5 h), PIDF-3 (6 h), PIDF-4 (7 h), and PIDF-5 (8 h)), a new 2θ peak at 32.7° was observed. These results indicate that the crystal region of PIDF was not completely destroyed but changed into a small ordered crystal structure with the intensification of ball milling treatment [35]. This is similar to a previous report that planetary ball milling destroyed the crystal structure of cellulose [7].

#### 3.1.5. Morphology Analysis

SEM images (Figure 1e) show that the ball milling modification changed the microstructure of PIDF to varying degrees. As can be seen from the 500× magnification, the five PIDF particles all had a spherical structure. With the extension of milling time, PIDF showed smaller spherical particles, consistent with particle size analysis (Figure 1a). The particles of PIDF-1 were large. In PIDF-2 and PIDF-3, the large blocky particles were mixed with a small number of small particles. PIDF-4 and PIDF-5 had smaller particles; the smallest particle size was observed in PIDF-5. This phenomenon shows that the extension of milling time caused the large particles of PIDF to decompose into smaller particles. Similar irregularly spherical and decreasing particle size results were also reported for ball-milled citrus fibers [11].

It was observed at 5000× magnification (Figure 1e) that, with the increase in milling time, the surface structure of PIDF became looser and coarser while the particle size decreased. The same results have been reported for disk-milled corn fiber [6], IDF from pomelo (*Citrus grandis*) peel [10], and grain bran [32]. Studies have shown that a decrease in particle size is generally associated with an increase in specific surface area and exposes more hydrophilic and hydrophobic groups, which may enhance the functional properties of dietary fiber, such as WHC, OHC, and WSC [10,36]. However, its chemical composition, such as of cellulose, hemicellulose, and lignin, will not change due to changes in particle size [11]. Furthermore, the finer particles appear to aggregate (PIDF-4 and PIDF-5) [30].

### 3.2. Functional Properties Analysis

#### 3.2.1. Effects of Ball Milling on Hydration Properties and OHC

**Figure 2:**
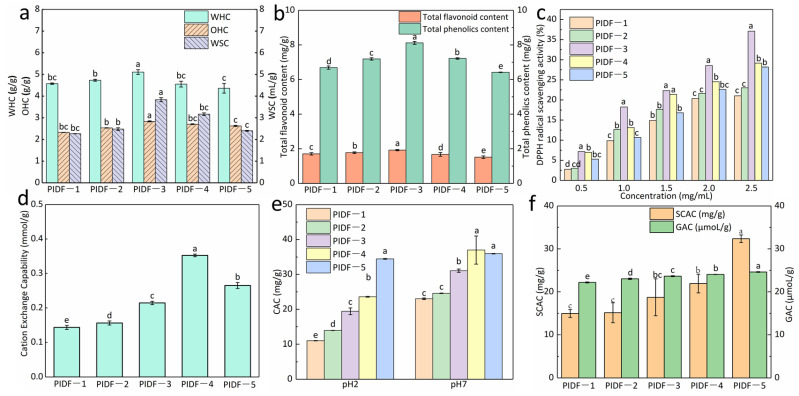
Functional properties of milled PIDF. (**a**): WHC, OHC, and WSC; (**b**): TFC and TFC; (**c**): Antioxidant ability; (**d**): CEC; (**e**): CAC, (**f**): SCAC and GAC). Different superscripts within the same properties indicate a significant difference (*p* < 0.05).

WHC, WSC, and OHC are related to dietary fiber structure and are crucial functional properties in food processing and in healthy food [37]. WHC and OHC quantify the ability of dietary fiber to retain water and oil, respectively. WSC causes the dietary fiber to affect fecal bulking [6]. The effects of ball milling on WHC, WSC, and OHC are shown in Figure 2a. As shown in Figure 2a, with the increase of ball milling time, the WHC, WSC, and OHC of PIDF increased and gradually dropped. When the milling time was 6 h (PIDF-3), WHC, WSC, and OHC, all reached their highest values, which were 5.12 ± 0.11 g/g, 3.83 ± 0.09 mL/g, and 2.83 ± 0.02 g/g, respectively.

According to the literature, dietary fiber’s WHC and SWC are based on their hydrophilic groups (e.g., –OH) and porous network structures [38]. The intense mechanical processing of ball milling destroyed the internal structure of the dietary fiber, loosening the porous mesh structure and reducing the particle size of the PIDF (Figure 2a). Therefore, the specific surface area of the PIDF gradually increased. More hydrophilic groups were exposed, making it fully contact and combine with water. As a result, the WHC and WSC of dietary fiber increased. When the ball milling treatment exceeded 6 h, the WHC and WSC values decreased as the milling time continued to extend. This could be attributed to the destroyed dietary fiber’s matrix structure, reducing its hydration capacity with a particle size that is too small [35]. Similar results of a decrease in WHC and WSC with a long-term ball milling treatment were observed for okara IDF [30]. Excessively small particle size would cause particles to agglomerate, thereby reducing the water-binding capacity of dietary fiber [30].

The OHC is related to the surface structure and hydrophilic groups of dietary fiber [39]. Similarly, excessively long milling times (PIDF-4 (7 h) and PIDF-5 (8 h)) reduced the OHC of PIDF. This reduction can be ascribed to the destruction of PIDF’s porous structure during intense ball milling. This hindered PIDF’s absorption into the oil. Similar results were also observed in ball-milled insoluble corn fiber [6] and asparagus leaf fiber [18]. In addition, agglomeration between particles (Figure 1e) can also reduce the hydration capacity and OHC of PIDF [30].

#### 3.2.2. TPC and TFC Content

The contents of TPC and TFC of the five kinds of PIDF modified by different milling times are shown in Figure 2b. As can be seen from Figure 2b, the TPC and TFC in PIDF firstly increased and then decreased with the prolonged milling time. When the milling time was 6 h, the TPC and TFC of PIDF-3 were the highest, increasing from 6.69 ± 0.09 mg/g (ball-milled 4 h) and 1.71 ± 0.06 mg/g (ball-milled 4 h) to 8.12 ± 0.08 mg/g and 1.91 ± 0.03 mg/g, respectively. The increase in TPC and TFC content was mainly due to the decrease of PIDF particles and the enlargement of surface area, which improved the extraction rate of TPC and TFC. In addition, high-intensity milling destroys plant cell walls, helps to release internal polyphenols and flavonoids, and promotes the effective extraction of total phenols and flavonoids [18]. Similar increases in TPC or TFC in dietary fiber powder from asparagus leaf by-product [18], mushroom powder [40], and persimmon seed powder [28] were also reported.

When the milling time was 7 h and 8 h, the TPC and TFC in PIDF did not increase but decreased (Figure 2b). Some reports have shown that the TPC of green tea powder decreases with the decrease in particle size [41] and the TFC of Koehne fruit powder decreases [42] with micronization. The decrease in TPC and TFC was due to the degradation of polyphenol and flavonoid compounds caused by exposure to oxygen during prolonged milling processes.

#### 3.2.3. Antioxidant Ability of PIDF

Figure 2c shows the results of the DPPH assay. The results show that the inhibition of DPPH free radicals was dose-dependent and steeply sloped. The report of [43] indicated that IDF from male date palm flowers also contributed to the inhibition of DPPH radicals in a concentration-dependent manner. In addition, PIDFs have different TPC and TFC content (Figure 2b). The phenolic and flavonoid substances combined with PIDF cause PIDF to have good antioxidant activity. Therefore, IDF has the potential to be used as a natural antioxidant. Furthermore, the antioxidant capacity of PIDF was related to the content of phenols and flavonoids that it binds to. Figure 2b shows that PIDF-3 had the highest total phenol and flavonoid content. Therefore, PIDF-3 shows the strongest inhibition (*p* < 0.05).

#### 3.2.4. Cation Exchange Capability (CEC)

Modification treatment will destroy the covalent bond structure in IDF molecules, exposing more functional groups, such as carboxyl and hydroxyl groups, which are more conducive to cation exchange. As can be seen from Figure 2d, with the extension of milling time, the CEC of PIDF showed a trend of first increasing and then decreasing, among which the CEC of PIDF-4 was the best (*p* < 0.05), reaching 0.35 ± 0.0038 mmol/g. Longer milling time can result in a smaller particle size so that PIDFs are exposed to more carboxyl and hydrocarbon groups, thus improving the CEC. Similar observations on the effect of milling on CEC have been reported by [15]. When the milling time was extended to 8 h, the combined effect of long-term shear force and friction force further reduced the particle size of PIDF-5, and the structure’s long chain and side chain groups were damaged. In addition, the electrostatic interaction and aggregation tendency of PIDF particles were strengthened during the grinding process, which weakened the cation adsorption and exchange efficiency.

#### 3.2.5. Adsorption Capacity Analysis

CAC is an important indicator for assessing the hypolipidemic property of the PIDFs [44]. The effect of milling time on the CAC of PIDF is shown in Figure 2e. With the extension of milling time, the CAC of PIDF gradually increased, especially for PIDF-5, which had the highest CAC at pH 2 (*p* < 0.05). Ball milling modification resulted in a looser structure of PIDF and increased surface voids (Figure 1e), which provided more binding sites for PIDF to bind cholesterol [11]. The authors of [7] treated citrus fiber with xylanase and ball milling. The results showed that the porosity indicated by the fiber was proportional to the specific surface area, and the more porosity, the greater the CAC value of the fiber. The authors of [11] modified three citrus fibers by ball milling. The results showed that with the increase in ball milling time, the CAC of the three kinds of citrus fibers showed an increasing trend, which agrees with present studies. In addition, at pH 2, the CAC value of PIDF with five different milling times was lower than that at pH 7. Under acidic conditions, there are more hydrogen ions, and both fiber and cholesterol should have positive charges. There is electrostatic repulsion between the two, so CAC was reduced. A similar phenomenon was reported by [44].

Figure 2f shows the influence of milling times on the five PIDFs’ SCAC. The results showed that the SCAC of the five kinds of PIDF increased significantly with longer milling time (*p* < 0.05). When the milling time was 8 h, the SCAC of PIDF-5 was 32.35 ± 0.87 mg/g, which was 55.17% higher than that at 4 h milling (PIDF-1, 14.50 ± 0.92 mg/g). The variation tendency of the GAC was in accord with that of SCAC in this study. PIDF-5 has the highest GAC for the longer milling treatment time (Figure 2f). The GAC of PIDF was increased by ball milling. It has been reported that the particle size, charge density, and hydrophobic groups on the fiber surface affect IDF’s SCAC and GAC [32]. It can be seen from Figure 1a,b that PIDF-5 has the smallest particle size and the largest potential value.

### 3.3. Emulsification Characteristics of the Five Kinds of PIDF

#### 3.3.1. Contact Angle Analysis

**Figure 3:**
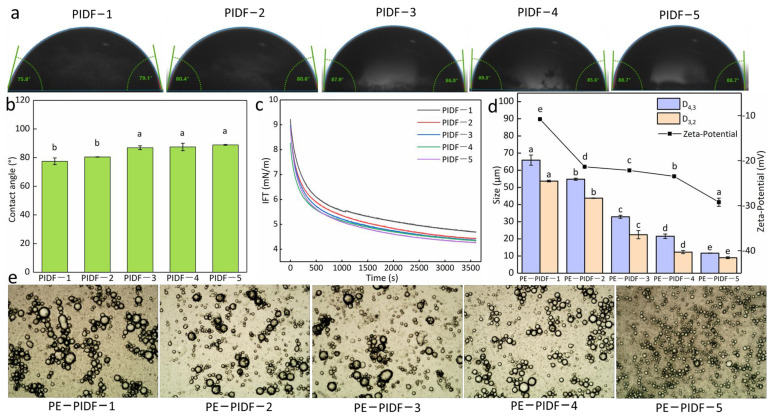
Emulsification characteristics of the five kinds of PIDF. (**a**,**b**): contact angle; (**c**): IFT, (**d**): droplet size and zeta-potential of Pickering emulsions; (**e**): optical microscope images of Pickering emulsions). Different superscripts within the same properties indicate a significant difference (*p* < 0.05).

The contact angle of a particle can reflect its wettability [23]. The particles with appropriate wettability can be stably adsorbed at the oil–water interface and used as solid emulsifiers to prepare Pickering emulsion [45]. When the contact angle is close to 90°, the particles show amphiphilicity and stable adsorption at the oil–water interface, preventing emulsion droplet agglomeration [46]. The contact angle of PIDF modified with different milling times is shown in Figure 3a,b. When the milling time increased, the contact angle of particles gradually increased. This was related to the reduction of particle size and the release of hydrophobic groups. The contact angle of all particles was less than 90°, indicating that PIDF can be used as an emulsifier for oil-in-water Pickering emulsion. When the grinding time was 8 h, the contact angle of PIDF-5 reached a maximum of 88.70° and was close to 90°, which was more suitable for use as a Pickering particle emulsifier. [10] also reported opposite changes in the contact angle of IDF from pomelo (*Citrus grandis*) peel after ball milling. According to another report, the contact angles of pomelo peel gradually decreased with increasing milling time [12]. In addition, the contact angle is affected by many factors, such as particle size, porosity, particle density, and the number of hydrophilic groups on the particle surface [23].

#### 3.3.2. IFT of PIDF

The dynamic adsorption behavior of PIDF at the oil–water interface was analyzed. The smaller the surface tension, the higher was the stability of the particle at the oil–water interface [12]. Figure 3c shows the IFT of the five kinds of PIDF. The results show that all PIDF samples’ IFT decreased rapidly and remained relatively stable over time, indicating the rapid spontaneous adsorption of fibers at the oil–water interface. The IFT of PIDF-1 was higher, which may be due to the larger particle size. With the increase in milling time, the particle size decreased, and the IFT of the particles also decreased. PIDF-4 and PIDF-5 had low surface tension, especially PIDF-5, which had the lowest surface tension when the adsorption reached stability. The results indicate that PIDF-5 has the best interfacial stability. Therefore, ball milling modification could be an effective method to reduce the interfacial tension of PIDF particles.

#### 3.3.3. Droplet Size and Zeta-Potential of Pickering Emulsions

The five kinds of PIDF were used to prepare Pickering emulsion, and the droplet size of emulsions is exhibited in Figure 3d. It could be observed that the droplet size decreased with increasing ball milling time. It is reported that the droplet size of the emulsion is the key property; the smaller the droplet size, the stronger the anti-coalescence ability, and the more conducive it is to maintaining the stability of the emulsion [47]. The droplet size stabilized by PIDF-5 (PE-PIDF-5) was smallest with the volume weighted mean (D_4,3_) at 11.66 ± 0.12 μm and surface weighted mean (D_3,2_) at 9.0 ± 0.53 μm. The smallest droplet size of the emulsion in PIDF-5 might be due to good wettability (Figure 3a,b) and small particle size (Figure 1a), which provided significant molecular coverage for the droplets and also modified the interface properties [48]. Similarly, the authors of [49] prepared the O/W Pickering emulsion using apple pomace nanoparticles, which showed that the droplet size of the emulsion gradually reduced from 12.9 μm to 550 nm with increments of milling time.

The zeta-potential could reflect the system’s stability, and the greater magnitude of charge determines the stability of the dispersed system [50]. Figure 3d shows the zeta-potential of emulsions prepared at various PIDFs and suggests that a longer ball milling time increases the absolute value of the zeta-potential. Similar results were reported by [10], showing that the Pickering emulsion stabilized by the insoluble dietary fiber of pomelo peel had greater absolute zeta-potential with the extension of ball milling time.

#### 3.3.4. Optical Microscope Images of Pickering Emulsions

The microscope images of the Pickering emulsions stabilized with the five kinds of PIDF are shown in Figure 3e. Larger droplets tend to coalesce, causing the emulsion to exhibit a creaming phenomenon, so the smaller the droplets, the higher the stability of the emulsion [48]. Figure 3e shows that the droplets of PE-PIDF-1, PE-PIDF-2, and PE-PIDF-3 emulsions were large in size and had an uneven distribution, and there were obvious droplet flocculation and aggregation phenomena. This instability may be due to the large droplet size, larger interfacial tension, and a lower zeta-potential value (Figure 3c,d). A lower zeta-potential value and reduced electrostatic repulsion will lead to droplet agglomeration and increased coalescence [51]. In contrast, PE-PIDF-4 and PE-PIDF-5 showed better performance, especially PE-PIDF-5, which has a smaller droplet size and more uniform distribution, which agrees with droplet size results. It could be reasonably concluded that the PIDF-5 dispersions have excellent emulsification ability. This means that the ball milling modification can help improve the application range of PIDF in Pickering emulsion.

#### 3.3.5. Rheological Properties

**Figure 4:**
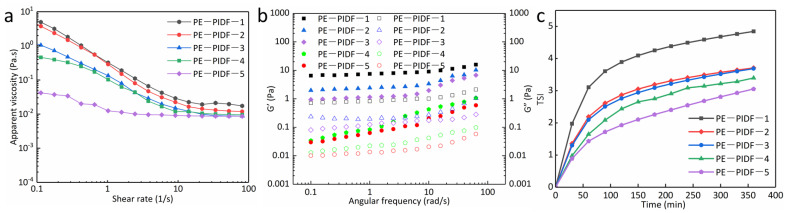
Rheological properties of Pickering emulsions. (**a**): apparent viscosity; (**b**): dynamic frequency sweep test, G′: closed symbols, G″: open symbols; (**c**): TSI.

The rheological properties of emulsions reveal the interactions between droplets and/or particles, which helps to determine the stabilization mechanism of emulsions [52]. Figure 4a shows the apparent viscosity results of the Pickering emulsion prepared by the five PIDFs. As shown in Figure 4a, with the increase in shear rate, the viscosity of the five emulsions gradually decreased, which is part of the shear thinning phenomenon [53]. PE-PIDF-5 showed the lowest viscosity at all shear rates. According to Figure 4b, the storage modulus G′ is always greater than the loss modulus G″ within the test frequency, exhibiting an elastic gel-like behavior [54]. Forming this gel-like network helps improve the emulsion’s stability. The research results of [8] showed that the apparent viscosity of Pickering emulsion prepared with IDF from pomelo peel modified by wet ball milling was low and had a gel-like behavior. The authors of [12] prepared a Pickering emulsion with ball-milled pomelo peel’s insoluble dietary fiber as a particle emulsifier. The rheological results demonstrated that the apparent viscosity of the Pickering emulsion decreased with the prolongation of milling time and had typical gel-like properties. The results reported above are consistent with the results of this study. The comparison indirectly indicates that PIDF-5 exhibits a potential to perform as a Pickering stabilizer. Therefore, ball milling modification can be used as a green modification of PIDF.

#### 3.3.6. Turbiscan Stability Index (TSI)

The TSI index can represent the stability of the overall dispersion system of the emulsion: the smaller the TSI index and the smaller the slope of the curve, the more stable the emulsion [55]. As shown in Figure 4c, the variation trend of the TSI index in the five kinds of Pickering emulsion prepared by PIDF with different particle sizes was the same. The TSI index gradually increased with time, and the stability worsened. The curve of the PE-PIDF-1 emulsion showed a rapid upward trend, which indicated the instability of PE-PIDF-1 emulsion in the initial stage. The curve of PE-PIDF-2, PE-PIDF-3, and PE-PIDF-4 emulsions showed an obvious upward trend in the initial stage, and the upward trend became gentle after 300 min. This suggests that the emulsion reached an equilibrium state with the increase of time, and the interaction between particles and the interfacial activity reached stability. The TSI index of PE-PIDF-5 emulsion was the lowest, and the curve showed a slow upward trend. This indicates that the PE-PIDF-5 Pickering emulsion has the best stability, and the intergranular interaction and interfacial activity are stable. The results are consistent with those of emulsion particle size and zeta-potential. The research results of [10,12] revealed that the prepared IDF of pomelo peel had a smaller particle size and was conducive to preparing a more stable Pickering emulsion.

#### 3.3.7. Storage Stability of Pickering Emulsions

**Figure 5:**
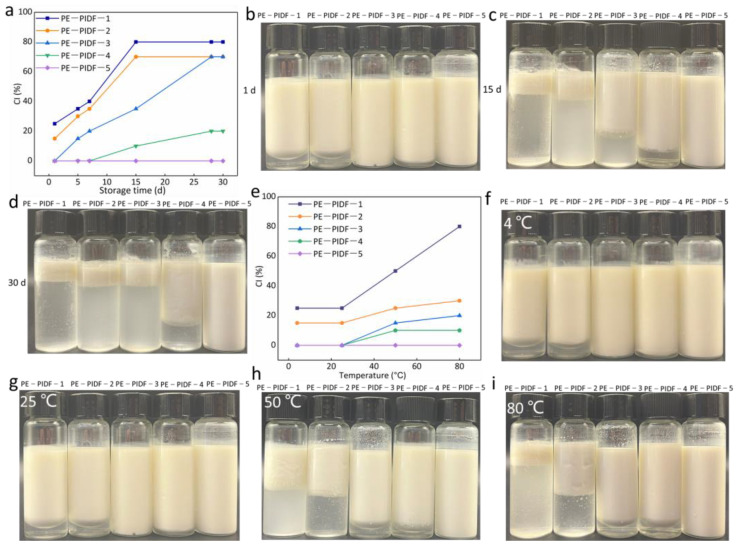
Storage stability and temperature stability of Pickering emulsions. (**a**): CI of the five Pickering emulsions during storage for 30 d; (**b**–**d**): photographs at 1, 15, and 30 d, respectively; (**e**): CI of the five Pickering emulsions at different temperatures; (**f**–**i**): photographs at 4, 25, 50, and 80 °C, respectively.

Storage stability reflects the coalescence degree of droplets and is a key characteristic of emulsion [56]. The CIs and images of all samples were monitored within 30 days. The results are shown in Figure 5a. In this study, the CIs of PE-PIDF-1 and PE-PIDF-2 increased significantly with increasing storage time, and a significant serum phase separation could be observed. In general, the turbidity of the serum layer can indicate the degree of droplet flocculation in the emulsion, and the more transparent the serum, the more severe the particle flocculation [56]. The visual results (Figure 5b–d) show that the transparent water layer formed by emulsification gradually decreased with the increase in milling time. This indicated that the stability of the emulsion decreased over time, possibly due to the larger particle size. The interaction forces between the particles (such as van der Waals forces) may be stronger, resulting in aggregation and stratification of droplets in the emulsion, reducing the emulsion’s stability. Compared with the first two emulsions, the anti-coagulation ability of PE-PIDF-3 increased. The CI value of PE-PIDF-4 increased first and then stabilized. The increase rate was significantly lower than the previous emulsions (PE-PIDF-1, PE-PIDF-2, and PE-PIDF-3). The trend of emulsion droplet aggregation was reduced to a certain extent (Figure 5d), thus improving the stability of the emulsion. There was no creaming of PE-PIDF-5 throughout the storage period. This excellent emulsifying performance is beneficial for its application in food-grade emulsion formulations. The particle size results show that the PE-PIDF-5 emulsion had the smallest droplet size (Figure 3d). The authors of [57] have also reported similar results for the storage stability of Pickering emulsion prepared by cellulose nanocrystals from coir fiber at 14 days, which suggests the reason is related to the droplet size reduction.

#### 3.3.8. Temperature Stability of Pickering Emulsions

The effects of temperature (4, 25, 50, and 80 °C) on the stability of PIDF-based Pickering emulsion were investigated. As shown in Figure 5e–i, the five kinds of PIDF-prepared Pickering emulsion have good stability at 4 and 25 °C, especially PE-PIDF-3, PE-PIDF-4, and PE-PIDF-5, which do not have creaming. The stability of PE-PIDF-1, PE-PIDF-2, PE-PIDF-3, and PE-PIDF-4 decreased with the increase in temperature, and the creaming was obvious. The authors of [58] prepared the Pickering emulsion using flexible cellulose nanofibrils. The results showed that high temperature would destroy the hydrogen bond between the cellulose, leading to emulsion instability. PE-PIDF-5 still has good stability at high temperatures (50 and 80 °C). This may be because PIDF-5 has the smallest particle size and has a larger surface area. Hence, it may require higher energy to break the stable structure of PE-PIDF-5. The authors of [50] also reported similar results in the stability of the Pickering emulsion prepared using pomelo spongy tissue cellulose nanofiber at high temperatures (80 °C) and suggested the reason was related to temperature, providing insufficient energy to desorb the cellulose from the oil–water interface.

#### 3.3.9. pH Stability of Pickering Emulsions

**Figure 6:**
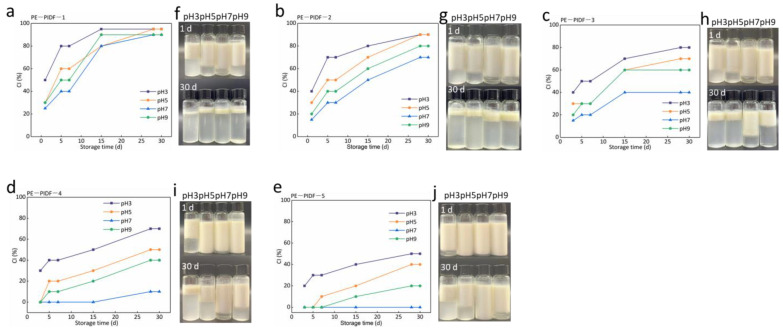
pH stability of Pickering emulsions. (**a**–**e**): CI of the five Pickering emulsions at pH 3, 5, 7, and 9, respectively; (**f**–**j**) photographs of the five Pickering emulsions at pH 3, 5, 7, and 9, respectively).

During the actual processing, there must be a change in pH value. Therefore, it is of practical significance to investigate the adaptability of PIDF-based Pickering emulsion to different pH values. Similarly, the appearance and CIs of Pickering emulsion prepared by 0.2% PIDF at pH 3, 5, 7, and 9 were analyzed. As shown in Figure 6, PE-PIDF-1, PE-PIDF-2, and PE-PIDF-3 emulsions have similarly changing trends and are unstable at different pH values. This was due to the short milling time of PIDF-1, PIDF-2, and PIDF-3 (4, 5, and 6 h, respectively), large particle size (Figure 1a), and a low absolute value of zeta-potential on the surface of the particles (Figure 1b), which cannot be firmly adsorbed at the oil–water interface and was insufficient to prepare stable Pickering emulsion. The stability of PE-PIDF-4 and PE-PIDF-5 emulsions was poor in an acidic environment, and the creaming phenomenon was obvious, which agreed with the reported result of [59]. In particular, the emulsions stabilized by PIDF-5 showed no creaming throughout the entire storage period of 30 days. In addition, PE-PIDF-4 and PE-PIDF-5 emulsions showed creaming in alkaline environments. The authors of [56] prepared the Pickering emulsion using citrus cellulose, and the results showed that when pH increased from 2 to 6, the zeta-potential value of the emulsion decreased, which helped maintain the emulsion’s stability. Figure 3d results show that PE-PIDF-5 has the largest absolute value of zeta-potential, which explains the stability of the emulsion in a neutral environment.

## 4. Conclusions

This study investigated the effects of ball milling treatment on the physicochemical properties, functional performance, and emulsification characteristics of PIDF. Ball milling was effective in decreasing the particle size of PIDF. The five kinds of PIDF all have a micrometer size. In particular, PIDF-5 has the smallest particle size and a looser and coarser microstructure. With the increase in milling processing time, there was an increase in zeta-potential value and similarly FTIR and XRD spectra. Ball milling treatment effectively promotes the functional properties (WHC, OHC, WSC, antioxidant ability, CEC, CAC, CASC, and GCA) and emulsifying capacity of PIDF. Ultimately, PIDF-5 could accumulate at the oil–water interface as a barrier film and stabilize the Pickering emulsion. In conclusion, ball milling treatment could be a promising method to produce high-quality PIDF. Further work will focus on underlining the application of PIDF-based Pickering emulsion in the food industry, which could facilitate the design of PIDF-based Pickering emulsion with specific application properties.

## Data Availability

The original contributions presented in the study are included in the article, further inquiries can be directed to the corresponding author.
